# Application of high-flow oxygen therapy in acute pancreatitis complicated with acute respiratory dysfunction

**DOI:** 10.55730/1300-0144.5364

**Published:** 2022-02-27

**Authors:** Xiang JI, Jie ZHOU, Wenting WU, Yan TANG, Tongrong XU

**Affiliations:** Department of Intensive Care Unit, Changzhou NO.2 People’s Hospital, Nanjing Medical University, Changzhou, China

**Keywords:** High-flow oxygen through nasal cannula, acute pancreatitis, respiratory dysfunction, acute respiratory distress syndrome

## Abstract

**Background/aim:**

To reveal the potential efficiency of high-flow oxygen therapy in acute pancreatitis complicated with acute respiratory dysfunction compared with conventional oxygen therapy.

**Materials and methods:**

We retrospectively analyzed 69 patients treated with high-flow oxygen or conventional oxygen therapy, then compared the difference of prime and second outcomes between the two groups.

**Results:**

The high-flow oxygen group had lower intubation rate (25.6% vs. 56.7%, p = 0.013) and longer median time to intubation (64.25 h vs. 7.75 h, p < 0.001) compared with the conventional oxygen group. High-flow oxygen had a stronger effect on improving dyspnea (87.2% vs. 56.7%, p = 0.006) and regression of respiratory failure (66.7% vs. 26.7%, p = 0.001). In the univariate and multivariate analyses, high-flow oxygen and APACHE II score were independent predict factors to respiratory failure regression (OR = 20.381, p = 0.038; OR = 36.827, p = 0.026). Patients treated with high-flow oxygen had shorter intensive care unit stay length (19.5 ± 13.4 vs. 7.8 ± 4.7, p = 0.009) and early mortality tended to be significantly lower (17.9% vs. 40.0%, p = 0.058).

**Conclusion:**

High-flow oxygen is a more effective method for acute pancreatitis complicated with acute respiratory dysfunction than conventional oxygen therapy.

## 1. Introduction

Pulmonary complications of acute pancreatitis (AP) characterized by increased permeability of the pulmonary microvasculature and alveolar spaces filled with leaked protein-rich exudate were frequent with morbidity about 75% [1,2], including acute lung injury (ALI) or ARDS, atelectasis, pleural effusion, alteration in diaphragmatic function, i.e. [3–5]. It usually resulted in a need for endotracheal intubation and mechanical ventilation, in addition, the mechanisms were complex and still to be discovered [6]. It was reported that 30%–60% of death was related to pancreatitis-associated ALI and ARDS [7–9]. Furthermore, nearly one-third of death of acute pancreatitis prior to admission to hospital were associated with ALI [10]. Studies have revealed that acute respiratory dysfunction (ARD) was an independent prognostic factor for hospital mortality in severe acute pancreatitis (SAP) along with the age, chronic health situation, and organ failures, which was associated with 60% of death within the first week [11,12]. Therefore, more attention has been paid to the treatment of pancreatitis-associated respiratory complications for reducing early death.

High-flow oxygen through nasal cannula (HFNC), which is increasingly used in intensive care unit (ICU) and non-ICU wards was considered as an alternative to noninvasive positive pressure ventilation (NPPV) and conventional oxygen therapy (COT) with better tolerated and decreasing work of breathing [13,14]. It could provide heated and humidified air and reduce airway secretion and atelectasis with high-flow rates of up to 60 L/min as well as high FiO2 from 0.21 to 1.0 [15,16]. Previous studies have revealed that HFNC has the capability of generating low levels of positive end-expiratory pressure and decreasing physiological dead space through flushing expired carbon dioxide into the upper airway [17]. It has been applied to various diseases, including various respiratory failure, cardiogenic pulmonary edema, postextubation, postoperative patients, and infants [15, 16, 18–20]. However, the efficiency of HFNC in acute pancreatitis is unclear. Our research is intended to illustrate the value of HFNC in acute pancreatitis complicated with acute respiratory failure.

## 2. Materials and methods

### 2.1. Trial design

All acute pancreatitis complicated with ARD admitted to ICU were included in this retrospective study from January 2014 to June 2019. This trial was approved by the ethical committee of our hospital (approval number: KY030-01) and was registered on the Chinese Clinical Trial Registry (www.chictr.org.cn/, registration number: ChiCTR2000029202), and written informed consent was obtained from patients or their families before HFNC treatment.

### 2.2. Patients

Computed tomography (CT) combined with supportive specific laboratory data were used in the diagnosis of AP. All AP were enrolled if they followed one of the following criteria: PaO2/FiO2 < 300 mmHg; respiratory rate > 25/min; dyspnea or accessory muscle use; asynchronous or paradoxical breathing; required > 5 L/min O2 to maintain SpO2 > 92%. Exclusion criteria: the need for immediate intubation, central nervous system disorder, cannot answer questions, contraindications for the use of the mask (noncooperative patient). Patients were asked to grade their dyspnea score [21] (improvement; no change; deterioration) and comfort score [22] (poor; acceptable; good) 1 h after the intervention.

### 2.3. Study intervention

All patients were treated with COT through a face mask or nasal cannula before enrolling. In the COT group, oxygen therapy was applied continuously through a face mask or nasal cannula at a flow rate of up to 10 liters per minute. The rate was adjusted to obtain SpO2 > 92% until patients recovered or were intubated. High-flow humidified oxygen (37 °C and 44 mgH2O/L) was delivered continuously through a nasal cannula with Optiflow (Fisher and Paykel Healthcare) with a primal flow rate of 50 L/min, a primal FIO2 of 50%, and dynamic adjustments to obtain SpO2 > 92%. HFNC was switched to COT if SpO2 was ≥ 95% at ≤ 5 L/min O2 or the PaO2:FIO2 was at least 300.

Intubation and mechanical ventilation decisions were made by the physicians with the criteria: respiratory arrest; respiratory pauses with loss of consciousness or gasping respiration; encephalopathy; cardiovascular instability; unmanageable secretions; respiratory fatigue; refractory hypoxemia (HFNC: SpO2 ≤ 88% with FIO2 = 100%; COT: SpO2 ≤ 88% with at least 10 L/min), or respiratory acidosis (pH < 7.30 and PaCO2 ≥ 50 mmHg).

### 2.4. Primary and second outcomes

The primary outcomes are the early intubation rate, defined as a percentage of intubation and mechanical ventilation in 10 days and the median time to intubation from patients enrolled. The second outcome includes changes in physiological parameters as well as arterial blood gases and grade of dyspnea, comfort score, regression of respiratory failure after 1-h intervention, adverse events (atelectasis, pleural effusion, and abdominal distension), early mortality defined as mortality in 10 days and ICU stay length.

## 3. Statistical analysis

The chi-square test or Fisher test was used in the comparisons for categorical variables while the unpaired student’s t-test or Mann-Whitney U test was performed for continuous variables. The analysis of the data obtained before and after intervention from each patient was made with the paired Wilcoxon test. Variables associated with respiratory failure regression were assessed by univariate and multivariate logistic-regression analyses. Variables suspected to be associated with regression of respiratory failure with a P < 0.10 after univariate analysis were accounted into the multivariate analysis. All statistical analyses were performed using IBM SPSS, Version 19.0 (IBM Corp., Armonk, NY, USA) or GraphPad Prism version 6.0 (GraphPad Software Inc., San Diego, CA, USA). A p-value (two-tailed) < 0.05 was considered statistically significant.

## 4. Results

### 4.1. Patients

A total of eighty-one patients with AP were admitted to ICU. Seven were intubated immediately at admission and five did not meet the classification criteria at last. Ultimately, sixty-nine patients were included in this retrospective study. Thirty out of 69 were treated with conventional oxygen through nasal cannula or face mask, while 39 were treated with HFNC.

Baseline characteristics of patients, including demographic data, the severity of illness (APACHE II), etiology, the severity of AP (Ranson score and Balthazar score), and associated comorbidities were recorded in Table 1. Vital signs and arterial blood gases before and after enrollment (1 h) are collected in Table 2. We also evaluated the incidence of atelectasis and pleural effusion in the two groups by CT.

### 4.2. Primary outcome

**Figure 1:**
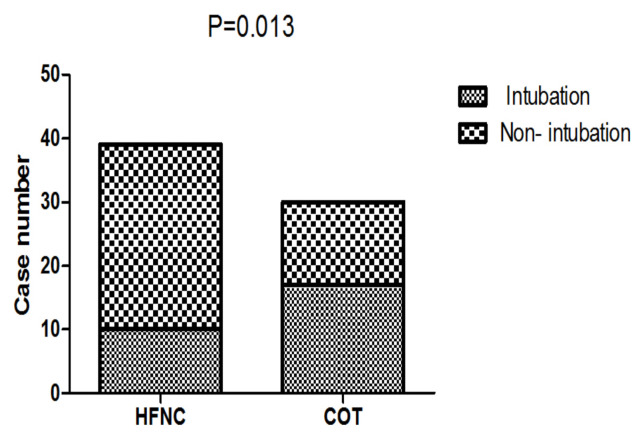
Comparison of intubation rate between the two groups. HFNC: High-flow oxygen though nasal cannula; COT: Conventional oxygen therapy

The early intubation rate was 25.6% (10 of 39 patients) in the HFNC group, 56.7% (17 of 30) in the COT group (p = 0.013), Table 3, Figure 1. The median time to intubation was 64.25h in the HFNC group, 7.75h in the COT group (p < 0.001). Table 3. Meanwhile, we compared the intraabdominal pressure (cmH2O) before intubation of intubated patients in the two groups (HFNC 21.7 vs. COT 19.1; p = 0.103; not shown).

### 4.3. Changes in physiological parameters and arterial blood gases

After 1 h of intervention, patients treated with HFNC had decreased respiratory rate (breaths/min), heart rate (beats/min) (27 vs. 21, p < 0.001; 121 vs. 104, p = 0.008) and increased PaO2 (mmHg) (64 vs. 110; p < 0.001), while PaCO2 (mmHg) and PaO2/FiO2 (mmHg) had no changes (37 vs. 42, p = 0.087; 189 vs. 210, p = 0.539) compared with baseline. However, in the COT group, after 1 h of intervention, respiratory rate, heart rate, and PaO2/FiO2 were similar (30 vs. 26, p = 0.166; 114 vs. 109, p = 0.486; 192 vs. 201, p = 0.142) with baseline, whereas PaCO2 and PaO2 were higher (38 vs. 50; p < 0.001; 64 vs. 83; p < 0.001), Table 2.

There was no difference in physiological parameters and arterial blood gases at baseline between the HFNC group and the COT group. Compared with the COT group, patients treated with HFNC had decreased respiratory rates, PaCO2 (21 vs. 26, p = 0.020: 42 vs. 50, p < 0.001) and increased PaO2 (110 vs. 83, p = 0.003) 1 h after intervention. Patients showed greater improvement in PaO2 in the HFNC group than that in the COT group (p < 0.001), Table 2.

### 4.4. Dyspnea grade, comfort score, and respiratory dysfunction regression

**Figure 2:**
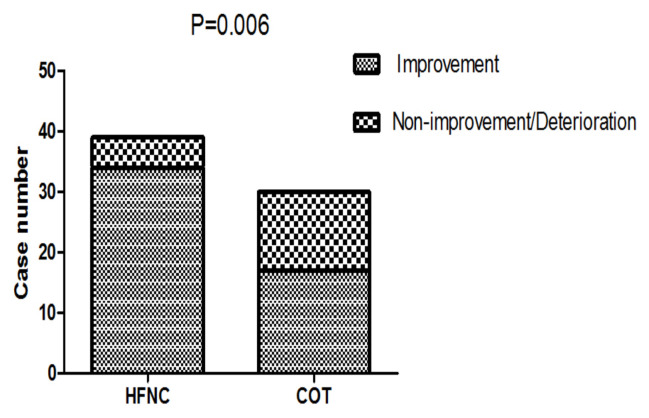
Comparison of dyspnea score improvement between the two groups 1 h after intervention. HFNC: High-flow oxygen through nasal cannula; COT: Conventional oxygen therapy

The comfort score was similar between the two groups (p = 0.596, Table 2). There was a higher proportion of patients feeling improvement in dyspnea in the HFNC group (87.2% vs. 56.7%, p = 0.006), Figure 2.

**Figure 3:**
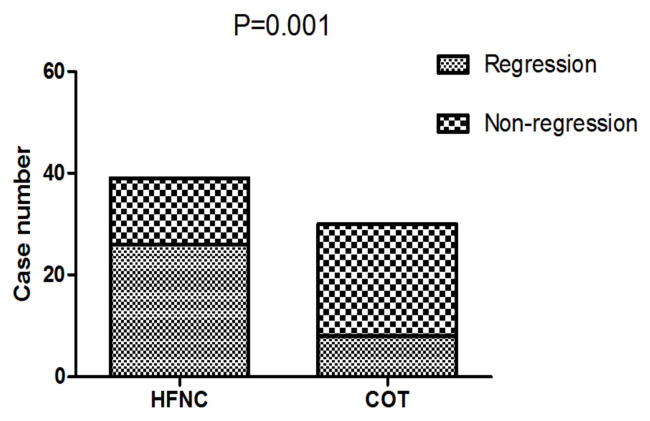
Comparison of respiratory failure regression between the two groups 1 h after intervention. HFNC: High-flow oxygen through nasal cannula; COT: Conventional oxygen therapy

Improved respiratory dysfunction was defined as respiratory rate <25/min and improvement in breathing effort, including accessory breathing muscle activity and/or paradoxical breathing. Finally, 34 out of 69 patients (26 in the HFNC group vs 8 in the COT group; p = 0.001; Figure 3) got respiratory failure regression 1 h after intervention. In the univariate analysis, the following parameters were associated with respiratory failure improvement: APACHE II score, PaO2 at baseline, and Oxygen strategies. Higher APACHE II score, lower PaO2 at baseline and conventional oxygen therapy exhibited an increased risk for poorer regression of respiratory failure (OR = 12.250, 95% CI 1.268–118.361, p = 0.030; OR = 8.000, 95% CI 1.252–51.137, p = 0.028; OR = 6.429, 95% CI 1.026–40.261, p = 0.047). In multivariate logistic-regression analyses, HFNC, and APACHE II score were independent predict factors to regression of respiratory failure (OR = 20.381, 95% CI 1.177–351.911, p = 0.038; OR = 36.827, 95% CI 1.529–887.083, p = 0.026), Table 4.

### 4.5. Adverse events and clinical outcomes

No significant differences were found for adverse events between the two groups. Early mortality in the HFNC group had a trend to be significantly lower than that in the COT group (17.9% vs. 40.0%, p = 0.058). Patients treated with COT had much longer ICU stay length (19.5 ± 13.4 vs. 7.8 ± 4.7, p = 0.009), Table 5.

## 5. Discussion

Accumulating research have revealed some advantages of HFNC application in pneumonia-associated respiratory dysfunction, including decreased intubation rate, lower 90-day mortality, and increased ventilator-free days compared with NPPV and/or COT [23–26]. Despite this, COT with a face mask or nasal cannula is still the first-line treatment for those lacking studies about HFNC application in AP-associated respiratory dysfunction. However, COT is not able to satisfy some patients with severe hypoxemia. Therefore, to our knowledge, this study illustrated the efficiency of HFNC in acute pancreatitis complicated with acute respiratory dysfunction for the first time. In this study, eliminating the effect of secondary infection on intubation, we compared the early intubation rate and the median time to intubation of the two groups in combination with the pathophysiological characteristics of pancreatitis. Severe acute pancreatitis has two representative phases. The first stage is in the first ten days, with characteristics of the systemic inflammatory response syndrome (SIRS). The second usually comes up during the second week and is marked by infectious manifestations [27]. In the end, we found patients in the HFNC group had a lower early intubation rate and a longer median time to intubation.

We also compared other outcomes, including changes in physiological parameters and arterial blood gases, comfort score, regression of dyspnea and respiratory dysfunction, adverse events, early mortality, and ICU stay length between the two groups and found that first, HFNC was superior to COT in improving dyspnea, decreasing respiratory rate and heart rate, preventing carbon dioxide retention, and had a stronger effect on improving PaO2. However, we did not find a difference in PO2/FiO2 between the two groups, indicating that HFNC could improve PO2 with higher FiO2, but it could not improve physiopathologic changes. Second, HFNC was independently associated with the regression of respiratory dysfunction. Third, the comfort score of HFNC was similar to that of COT. At last, patients treated with HFNC had shorter ICU stay length and lower early mortality. We hypothesize that the reason why the early mortality between the two groups did not reach statistical significance would be due to the limited number of patients in our study. Above all, we would come to the conclusion that HFNC is prior to COT in acute pancreatitis complicated with acute respiratory dysfunction. There are some limitations to our reach. First, this study is a single-center with a small number of patients. Second, we did not compare HFNC and NPPV. Hence, in the future, we should compare HFNC, NPPV, and COT in multicenter studies with large samples.

## Figures and Tables

**Table 1:** Patients characteristics.

Characteristics	HFNC (n = 39)	COT (n = 30)	P
Age, mean, y-no. (%)		0.332	
<52	18(46)		18(60)
≥52	21(54)		12(40)
Gender-no. (%)		0.09	
Men	19(49)		21(70)
Female	20(51)		9(30)
Etiology of AP-no. (%)		0.285	
Alcoholic	6(15)		6(20)
Biliary	23(59)		12(40)
Hyperlipidemic	10(26)		12(40)
Ranson score at admission		0.809	
<3	20(51)		14(47)
≥3	19(49)		16(53)
Ranson (48 h) score		1.000	
<3	19(49)		15(50)
≥3	20(51)		15(50)
Balthazar score		0.472	
≤6	22(56)		14(47)
>6	17(44)		16(53)
APACHE II score, mean-no. (%)		0.631	
<10	18(46)		16(53)
≥10	21(54)		14(47)
SOFA score, mean-no. (%)		0.806	
<5	15(38)		13(43)
≥5	24(62)		17(57)
Current or past smoking-no. (%)		0.621	
Yes	14(36)		13(43)
No	25(64)		17(57)
Associated comorbidities-no. (%)		0.865	
Arterial hypertension	13(50)		9(43)
Diabetes	7(27)		7(33)
Cardiac insufficiency	6(23)		5(24)

HFNC: High-flow oxygen through nasal cannula; COT: Conventional oxygen therapy

**Table 2:** Physiological parameters and arterial blood gases at baseline and 1 h after intervention.

Parameters	Mean (95% CI)								
	Baseline			1 h					
	HFNC	COT	P	HFNC	COT	P	P^a^	P^b^	P^c^
Heart rate, beats/min	121 (111–130)	114 (107–121)	0.286	104 (99–110)	109 (99–118)	0.417	**0.008**	0.486	**0.023**
Respiratory rate, breaths/min	27 (25–29)	30 (26–33)	0.379	21 (19–22)	26 (23–29)	**0.020**	**<0.001**	0.166	**0.011**
PaCO2, mmHg	37 (34–40)	38 (35–42)	0.573	42 (40–44)	50 (45–55)	**<0.001**	0.087	**<0.001**	0.053
PaO2, mmHg	64 (62–65)	64 (60–67)	0.789	110 (99–121)	83 (79–86)	**0.003**	**<0.001**	**<0.001**	**<0.001**
PaO2/FiO2 mmHg	189 (170–208)	192 (165–219)	0.628	210 (179–240)	201 (182–220)	0.561	0.539	0.142	0.856
Comfort score, No. (%)						0.596			
Poor				6(15.4)	5(16.7)				
Acceptable				12(30.8)	6(20.0)				
Good				21(53.8)	19(63.3)				

HFNC: High-flow oxygen through nasal cannula; COT: Conventional oxygen therapy;

a Baseline vs. 1 h in HFNC;

b Baseline vs. 1 h in COT;

c Changes in the HFNC group vs. that in the COT group 1 h after the intervention.

**Table 3:** Primary outcome.

Parameters	HFNC	COT	P
Intubation number no. (%)	10(25.6)	17(56.7)	**0.013**
Median time to intubation median ± SD, h	64.25 ± 12.71	7.75 ± 2.96	**<0.001**

HFNC: High-flow oxygen through nasal cannula; COT: Conventional oxygen therapy

**Table 4:** Univariate and multivariate analysis of risk factors to regression of respiratory failure.

Variables	Univariate analysis			Multivariate analysis		
	Odds ratio	IC (95%)	P	Odds ratio	IC (95%)	P
Age, mean, y			0.165			
<51 ≥51	3.6	0.591–21.932				
Gender			0.698			
Men/Female	0.720	0.137–3.784				
Etiology of AP						
Alcoholic			0.157			
Biliary	3.250	0.163–64.614	0.440			
Hyperlipidemic	0.208	0.017–2.518	0.217			
Ranson score at admission			0.113			
<3, ≥3	4.333	0.708–26.531				
Balthazar score			0.301			
≤6, >6	2.407	0.456–12.720				
APACHE II score, mean			**0.030**	36.827	1.529–887.083	**0.026**
<10, ≥10	12.250	1.268–118.361				
SOFA score, mean			0.954			
<5, ≥5	1.050	0.197–5.602				
Current or past smoking	1.050	0.197–5.602	0.954			
Clinical parameters at baseline, median						
Heart rates, beats/min			0.825			
<116, ≥116	1.200	0.237–6.065				
Respiratory rate, breaths/min			0.301			
<29, ≥29	0.415	0.079–2.195				
Arterial blood gas at baseline, median				6.275	0.165–4.212	0.149
PaCO2, mmHg	0.833	0.518–75.960	0.825			
<38, ≥38						
PaO2, mmHg			**0.028**			
<63, ≥63	8.000	1.252–51.137				
PaO2/FiO2, mmHg			0.698			
<181, ≥181	1.389	0.264–7.299				
Oxygen strategies, no. (%)			**0.047**	20.381	1.177–351.911	**0.038**
High-flow oxygen/Conventional oxygen therapy	6.429	1.026–40.261				

**Table 5:** Adverse events and clinical outcomes.

Events	Group		P
	COT (n = 30)	HFNC (n = 39)	
Atelectasis-no. (%)	24(80.0)	30(76.9)	1.000
Pleural effusion-no. (%)	22(73.3)	26(66.7)	0.606
Abdominal distension-no. (%)	12(40.0)	14(35.9)	0.804
Early mortality-no. (%)	12(40.0)	7(17.9)	**0.058**
ICU stay length, median ± SD, d	19.5 ± 13.4	7.8 ± 4.7	**0.009**

HFNC: High-flow oxygen through nasal cannula; COT: Conventional oxygen therapy
